# Gut–Brain Axis and Psychopathology: Exploring the Impact of Diet with a Focus on the Low-FODMAP Approach

**DOI:** 10.3390/nu16203515

**Published:** 2024-10-17

**Authors:** Emanuela Ribichini, Giulia Scalese, Chiara Mocci, Carola Severi

**Affiliations:** Department of Translational and Precision Medicine, Sapienza University of Rome, 00161 Rome, Italy; giulia.scalese@uniroma1.it (G.S.); chiara.mocci@uniroma1.it (C.M.); carola.severi@uniroma1.it (C.S.)

**Keywords:** gut brain axis, irritable bowel syndrome, gut microbiota, psychopathology, depression, anxiety, low FODMAP diet

## Abstract

Background: The gut–brain axis (GBA) is a bidirectional communication network connecting the central nervous system with the gastrointestinal (GI) tract, influencing both mental and physical health. Recent research has underscored the significant role of diet in modulating this axis, with attention to how specific dietary patterns can impact anxiety and depression, particularly when linked to disorders of gut–brain interaction (DGBIs), like intestinal bowel syndrome (IBS). Aims and Methods: This narrative review examines the effects of specific diet regimens on the GBA and its potential role in managing psychopathology, focusing on anxiety and depression, IBS, and the low-FODMAP diet. We conducted a search on PubMed and MEDLINE by combining the following key terms: “Gut–Brain Axis”, “Irritable Bowel Syndrome”, “Low FODMAP diet”, “Mediterranean Diet”, “Psychopathology”, “Anxiety and Depression”, and “Gut Microbiota”. We applied the following filters: “Clinical Trials”, “Randomized Controlled Trials”, “Reviews”, “Meta-Analyses”, and “Systematic Reviews”. In total, 59 papers were included. Results: Low-FODMAP diet, originally developed to alleviate GI symptoms in IBS, may also positively influence mental health by modulating the GBA and improving the gut microbiota (GM) composition. New insights suggest that combining the low-FODMAP diet with the Mediterranean diet could offer a synergistic effect, enhancing both GI and psychological therapeutic outcomes. Conclusions: Understanding the complex interactions between diet, the GM, and mental health opens new avenues for holistic approaches to managing psychopathology, particularly when linked to GI symptoms.

## 1. Introduction

The gut–brain axis (GBA) is a complex communication network connecting the gastrointestinal (GI) tract with the central nervous system (CNS). Its role is to monitor and integrate gut functions as well as to link emotional and cognitive centers of the brain with peripheral intestinal functions and mechanisms such as immune activation, intestinal permeability, enteric reflex, and enteroendocrine signalling. This bidirectional system encompasses neural, hormonal, immune, and microbial pathways, with the gut microbiota (GM) playing a crucial role in regulating these interactions [1]. Over the past decade, emerging evidence has significantly deepened our understanding of the intricate bidirectional relationship between the gut and the brain and clarified the important role of the GM.

Both clinical and experimental evidence suggest that the GM has an important impact on the GBA, interacting not only locally within the gastrointestinal tract but also directly with the CNS through neuroendocrine and metabolic pathways. Research demonstrates that the gut microbiota (GM) is not only essential for the development and healthy functioning of the mammalian brain but also plays a key role in various physiological processes [2]. These include the maturation of the immune system, the healthy functioning of the hypothalamic–pituitary–adrenal (HPA) axis and the endocrine system, the formation and maintenance of the blood-brain barrier, neurogenesis, and myelination [2].

Disruptions in GM composition—often referred to as dysbiosis—are linked to a wide range of neuropsychiatric disorders by affecting brain function and behaviour and potentially contributing to conditions such as anxiety, depression, autism spectrum disorders, and schizophrenia [3]. Evidence highlights distinct differences in GM composition between individuals with depression and healthy controls. For instance, studies have shown that individuals with depression typically exhibit reduced levels of beneficial bacteria like Lactobacillus, Bifidobacterium, Firmicutes, Faecalibacterium, and Ruminococcus, while levels of bacteria such as Provotella, Bacteroides, and Proteobacteria are elevated [4]. Even if it remains to be established whether depression symptoms lead to GM diversity changes or whether altered GM diversity contributes to psychopathological symptoms, several studies suggest that dysbiosis may play a causal role in depression-like behaviours [5,6]. For example, significant correlations have been identified between antibiotic use (in terms of dose and duration) and the likelihood of neuropsychiatric disorders, suggesting that antibiotics may trigger such conditions. Antibiotic exposure during the first year of life is associated with higher rates of behavioural problems, depressive symptoms, and neurocognitive decline later in life [7]. Further, other reports have documented psychiatric side effects, including anxiety and major depression, in patients receiving antibiotics, suggesting that dysbiosis can causally contribute to mental health disorders [7]. Preclinical research confirms that antibiotic-induced dysbiosis can provoke anxiety- and depression-like behaviours in rodents and faecal microbiota transplantation (FMT) from patients with depression into animal models induced depressive symptoms and metabolic changes [8,9].

The GBA framework also provides insight into the connection between psychiatric and GI disorders, with mood disorders such as anxiety and depression highly prevalent among patients with GI disorders like intestinal bowel syndrome (IBS) [10]. Similarly, individuals with anxiety or depressive disorders frequently report GI symptoms. Thus, chronic bowel symptoms, whether organic or functional, are associated with greater psychopathological severity than that in the general population. Symptoms affecting daily life contribute to higher stress levels, and it is widely recognized that up to 40–60% of IBS patients exhibit psychopathology, particularly anxiety, depression, panic, post-traumatic stress disorder, and somatization, compared with <25% in those with organic GI disorders and <20% in healthy controls [11]. For instance, patients with higher levels of anxiety or depression may experience less relief and lower quality of life (QoL) from standard IBS treatments compared to those with lower levels of psychological distress [12]. Piacentino D et al. [13] found that IBS patients show significantly higher global severity index (GSI), depression, and anxiety scores than those with (Inflammatory Bowel Disease) IBD, with greater disease severity linked to worse psychopathological outcomes in both groups. Additionally, patients with both IBS and functional dyspepsia (FD) show greater psychopathological symptom severity than those with IBS alone, corroborating the findings from Talley et al. [14] and Balboa et al. [15].

Dysregulation of the GBA may explain how gut disturbances impact brain homeostasis. Such dysregulation can result from direct intestinal damage (e.g., in GI diseases) or indirect effects mediated by the microbiota. Changes in the microbial community may affect the brain via various pathways, including metabolite production (e.g., butyrate with its anti-inflammatory properties), immune modulation, neurotransmitter regulation (e.g., serotonin, crucial for mood), and direct gut–brain communication via the vagus nerve and other neural pathways. Understanding and targeting the bidirectional relationship between the body and mind through the GBA is critical for effective treatment. As microbial metabolites and microbiota-driven neural signalling pathways are potential mechanisms through which gut dysbiosis may affect brain function and contribute to psychiatric symptoms, targeting the GM could offer a promising therapeutic strategy for depression and other neuropsychiatric disorders, aligning with the microbiota–GBA hypothesis [16].

Diet significantly influences the GBA, affecting both gut health and brain function, as well as the levels of psychopathology. Notably, the GM is profoundly influenced by both the quality and quantity of dietary intake, and in turn, this can affect mood and overall well-being. This bidirectional relationship underscores the crucial role of nutrition in shaping brain function and emotional well-being. As research continues to reveal, specific dietary patterns and nutrients can either support or alter the balance of the GBA, offering a potential therapeutic target for psychopathological conditions. The aim of this narrative review was to explore how dietary modulation of the GBA influences various forms of psychopathology, with a focus on anxiety, depression, and the potential mechanisms involved. The final section examines the impact of the low-FODMAP diet on these two different psychological conditions when associated with IBS.

## 2. Methods

This narrative review examines the effects of specific diet regimens on the GBA and its potential role in managing psychopathology, focusing on anxiety and depression, IBS, and the low-FODMAP diet. We conducted a search on PubMed and MEDLINE by combining the following key terms: “Gut–Brain Axis”, “Irritable Bowel Syndrome”, “Low FODMAP diet”, “Mediterranean Diet”, “Psychopathology”, “Anxiety and Depression”, and “Gut Microbiota”. We applied the following filters: “Clinical Trials”, “Randomized Controlled Trials”, “Reviews”, “Meta-Analyses”, and “Systematic Reviews”. The literature search was conducted from the year 2000, with the final MEDLINE search performed on 30 September 2024. Only articles in English were included, and no age limits were applied, although studies involving adult populations (18 years and above) were primarily analyzed. In detail, we included 59 papers, as follows: 12 review articles, 6 randomized clinical trials, 7 meta-analyses, 15 original research articles, 10 observational studies, 5 case reports/case series, and 4 others (guidelines or recommendations, letters to the editor, abstracts, and editorials).

## 3. Dietary Modulation of the Gut–Brain Axis and Its Influence on Psychopathology

**Figure 1:**
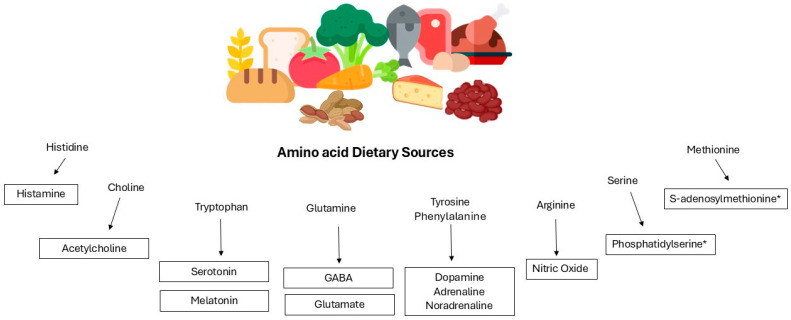
Overview of dietary amino acid precursors and their corresponding neurotransmitters involved in mental health and neurobiology. Abbreviation: GABA, gamma-aminobutyric acid. * serve as neurotransmitter modulators.

In recent years, there has been a growing interest in identifying therapeutic strategies that improve mood by regulating the composition of the intestinal microbiome, and the modulation of the GBA through diet is an emerging area of research, exploring how dietary patterns and components can influence the GM and, consequently, impact brain function and mental health. Numerous studies suggest that this relationship might be driven by the effects of specific dietary components on the expression of the GM [17]. One possible mechanism involves the release of neuroactive substances by specific amino acid precursors, critical for brain function, regulating mood, attention, and stress responses within the nervous system (Figure 1).

For example, protein consumption slows carbohydrate absorption and increases the release of dopamine and norepinephrine, both of which have a direct impact on mood [18]. Similarly, carbohydrate consumption increases serotonin levels, which also positively affects mood [18]. In general, the ways in which diet impacts anxiety and depression likely involve multiple interconnected pathways, both direct and indirect. These include mechanisms related to the immune response, oxidative stress, the gut microbiome, brain plasticity, neurotransmitter synthesis, and mitochondrial function [19].

The pilot study by Sydney E. Martin et al. [18] examined the dietary nutrient composition, mood, happiness, and GM of 20 patients. The study found that dietary changes can affect mood and happiness, with higher fat and carbohydrate intake being directly associated with increased anxiety and depression and inversely related to gut microbiome diversity. Conversely, a diet higher in fat and protein was linked to reduced anxiety and depression [18]. Gut bacteria also play a role in neurotransmitter metabolism, converting dietary tryptophan into 5-hydroxytryptamine (5-HT) via enterochromaffin cells [20]. Other microbes produce neurotransmitters such as norepinephrine and dopamine, which interact with the enteric nervous system or stimulate vagal sensory neurons in the gut, leading to brain activation and the regulation of homeostatic and reward-based feeding behaviour [21,22].

Based on this panorama, there is growing preclinical and clinical research on the influence of dietary habits and dietary interventions on various psychiatric and neurological disorders, including depression, cognitive decline, Parkinson’s disease, autism spectrum disorder, and epilepsy [23]. One such intervention involves omega-3 polyunsaturated fatty acids (PUFAs), particularly docosahexaenoic acid (DHA), eicosapentaenoic acid (EPA), and omega-6 (*n*-6) PUFAs like arachidonic acid (AA). Research has shown that levels of these PUFAs are reduced in individuals suffering from anxiety and depression [24,25]. Supplementation with these fatty acids has been found to alleviate the symptoms of these conditions, potentially by modulating the composition of the GM [26].

However, diet involves much more than simply supplementing nutrients or taking dietary supplements, and the role of diet in influencing mental health and psychopathology has gained significant attention in recent years. To note, emerging evidence suggests that what we eat can have profound effects on brain function, mood regulation, and the development or exacerbation of psychiatric disorders. Poor dietary habits, particularly diets high in processed foods and low in essential nutrients, have been associated with an increased risk of depression and anxiety [27].

A balanced diet rich in complex food matrices, diverse dietary fibers, phytochemicals, and live bacteria promotes the growth of beneficial gut microbes. These microbes, in turn, produce neuroactive compounds and health-promoting metabolites, such as short-chain fatty acids (SCFAs) [28]. SCFAs stimulate enteroendocrine L cells to release appetite-regulating hormones like glucagon-like peptide-1 (GLP-1) and peptide YY (PYY), which influence the hypothalamic centers that control eating behaviour [29]. Additionally, SCFAs support intestinal barrier integrity and enhance the immune system by modulating cytokine production from myeloid cells and promoting the differentiation of T regulatory and T helper cells, contributing to overall mental well-being [30].

In contrast, a diet high in processed foods, low in fiber, and rich in saturated fats, salt, and additives can negatively impact gut health, which in turn contributes to psychopathology [4]. This type of diet reduces microbiome diversity and disrupts bile acid metabolism while also decreasing mucus-producing bacteria, thereby weakening the gut barrier and loosening tight junctions [31]. The result is often the release of inflammatory markers and the translocation of endotoxins from the gut into the bloodstream, triggering low-grade systemic inflammation, linked to mental health disorders and impaired metabolic function [4].

Further evidence of the influence of diet on mental well-being came from randomized controlled trials (RCTs). The PREDIMED trial investigated the impact of the Mediterranean diet (MD) on depression, particularly in individuals at high cardiovascular risk. The trial was a randomized controlled study that primarily aimed to assess the effects of MD on cardiovascular health, but also explored its influence on mental health, including depression [32]. The participants were divided into groups that followed a MD supplemented with either extra-virgin olive oil or nuts and a control group that followed a low-fat diet. The results suggested that individuals who followed the MD, especially those with a higher adherence to it, showed a reduced risk of depression compared to those in the control group. The protective effects were more pronounced in participants with type 2 diabetes (multivariate HR = 0.59; 95% CI 0.36 to 0.98) [32]. The SMILES trial was a 12-week randomized controlled study designed to evaluate the effect of a dietary intervention on moderate to severe depression [33]. The intervention was a modified MD based on the Australian dietary guidelines and the dietary guidelines for adults in Greece [34,35]. The primary outcome was depression symptom improvement, measured by the Montgomery–Åsberg depression rating scale (MADRS), after 12 weeks. The study included 67 participants (33 in the diet group and 34 in the control group), with 55 of them also receiving some form of therapy (psychotherapy, pharmacotherapy, or both). The intervention group received seven individual nutritional counselling sessions with a clinical dietician, while the control group received social support. The results showed that the dietary intervention group had a significantly greater reduction in depressive symptoms compared to the control group, with a Cohen’s d effect size of –1.16, indicating a large effect. Specifically, 32.3% of the diet group achieved remission (MADRS score < 10) compared to 8.0% in the control group [33]. These findings suggest that improving diet could offer an effective and easily accessible approach to treating depression, a widespread mental health condition. The benefits of such dietary interventions may also extend to addressing other common co-occurring health issues, making it a potentially valuable addition to traditional treatment strategies.

Three other studies evaluated the impact of the MD on psychopathology. The HELFIMED study aimed to determine whether a MD supplemented with fish oil could improve mental health in adults with depression [36]. Specifically, increased adherence to the MD, particularly through higher consumption of nuts and diverse vegetables, was associated with reduced depression. There was also a correlation between improved mental health and higher omega-3 levels alongside lower omega-6 levels [36].

Similarly, the PREDIDEP study is a two-year randomized trial that examined the effects of a MD supplemented with extra virgin olive oil on depression recurrence. Depressive symptoms were assessed at the start and after 4, 8, 16, 20, and 24 months using the Beck depression inventory. Results showed a significant improvement in depressive symptoms with MD compared to the control group [37].

More recently, Staudacher HM et al. [38] conducted a RCT to evaluate the feasibility of a MD for IBS and its impact on GI and psychological symptoms. Adults with Rome IV IBS and mild to moderate anxiety and/or depressive symptoms were recruited for a 6-week period. A total of 59 individuals were randomized (29 to the MD, 30 to the control group with a habitual diet). The Mediterranean diet adherence screener score (MEDAS) was significantly higher in the MD group compared to controls at week 6 (7.5 vs. 5.7) and showed a greater increase (2.1 vs. 0.5), indicating that the MD is feasible. A higher proportion of individuals in the MD group responded to GI symptoms (83% vs. 37%) and depression (52% vs. 20%) compared to the control group [38].

Finally, an interestingly Chinese meta-analysis focusing on the relationship between fermented dairy foods intake and depression suggested that consuming fermented dairy foods may offer potential benefits for reducing depression, possibly through the GBA [39].

In summary, accumulating evidence from these interventional trials underscores the significant role that dietary interventions play in modulating the GBA, with measurable impacts on mental health outcomes. By targeting inflammation, the GM, and metabolic pathways, these studies collectively demonstrate that adopting a nutrient-dense anti-inflammatory diet can serve as a promising adjunctive treatment for mood disorders, such as depression and anxiety. Table 1 provides a summary of studies on specific dietary interventions and their impact on anxiety and depression.

## 4. Low-FODMAP Diet as a Modulator of the Gut–Brain Axis and Its Impact on Psychopathology

In recent years, there has been growing interest in foods containing FODMAPs (Fermentable oligo-, di-, monosaccharides, and polyols). These compounds increase the small bowel water content, elevate gas production (notably hydrogen and methane), promote excessive SCFA production, and heighten small intestinal motility. For individuals with significant visceral hypersensitivity, these effects can lead to symptoms such as abdominal pain, bloating, flatulence, and altered bowel habits [40]. Restricting FODMAPs has resulted in significant symptom improvement in up to 80% of IBS patients, a finding consistently supported by recent meta-analyses that confirm the diet’s effectiveness in managing IBS symptoms [41,42].

Other evidence suggests that a low-FODMAP diet (LFD) shows promise in improving both physical and emotional well-being by significantly reducing GI symptoms and enhancing various aspects of QoL and mental health in IBS patients. This dietary approach was suggested already 20 years ago by Ledochowski et al. [43], who suggested that the ingestion of FODMAPs negatively affected mood and that the elimination of dietary FODMAPs improved depressive symptoms. Similarly, Piacentino et al. [44] demonstrated that the LFD significantly improved not only the intensity and frequency of bloating and abdominal pain but also the SCL-90-R GSI, anxiety, and phobic anxiety scores, both immediately post-diet and at follow-up [44]. Later, other evidence came from RCTs. Another trial by Eswaran et al. [45] compared LFD to standard dietary advice with outcomes assessing QoL, anxiety, depression, and sleep and showed significant improvements in all assessed outcomes after 4 weeks of follow-up. Staudacher et al. [46] compared LFD to a sham diet, assessing various aspects of QoL, with improvements noted in some aspects of QoL but not in overall QoL after 4 weeks of follow-up. Harvie et al. [47] confirmed an increase in QoL in a relatively small cohort of LFD with a follow-up period of 6 months. Conversely, Pedersen et al. [48], in a short-term (6-week) study comparing LFD to a probiotic treatment, found no significant difference in the improvement of IBS-SSS and QoL. Of note, Ustaoğlu T et al. [49] conducted a RCT on 52 female IBS patients with 6-week LFD or LFD plus a supplement containing 6 billion units of Lactabacillus rhamnosus GG. The results showed a significant reduction of the IBS-SSS, anxiety, and depression scores of the individuals in both groups and an increase of IBS-QOL, suggesting that the addition of probiotics may not confer further benefit beyond the LFD alone on these metrics.

More recently, Tim L. Kortlever et al. [50] confirmed that a LFD offers a broad range of well-being benefits for IBS patients beyond the improvement of GI symptoms. In addition to reducing common IBS symptoms like bloating, abdominal pain, and irregular bowel movements, the LFD contributed to better overall condition management. Patients following the diet also reported reduced fatigue, which enhanced their overall sense of well-being, along with improvements in anxiety, depression, happiness, and vitality.

Research suggests that adherence to a LFD may improve intestinal barrier function in individuals with IBS. Elevated levels of inflammatory markers, such as interleukin-6 (IL-6), IL-10, and lipopolysaccharides (LPS)—a marker of bacterial translocation—found in IBS patients, especially in those with diarrhea-predominant IBS (IBS-D), significantly decreased during the LFD period, which coincided with improvements in both GI symptoms and psychological states [51]. Similarly, Stevens et al. [52] observed correlations between increased intestinal permeability, altered faecal microbiota, and elevated LPS levels in patients with anxiety or depression, even in the absence of GI symptoms.

Furthermore, by reducing the intake of fermentable carbohydrates that can exacerbate symptoms and promote a low-grade immune activation [53,54], an LFD may help restore the integrity of the intestinal barrier, determining a concurrent improvement in clinical and psychological conditions. Supporting this idea, Prospero L. et al. [51] conducted a 12-week study on IBS-D patients following an LFD. They observed improvements in both GI symptoms and psychological well-being, alongside enhanced intestinal permeability and mucosal integrity (as measured by I-FBP and Zonulin) and reductions in markers of inflammation and dysbiosis. The study concluded that the effectiveness of the LFD in improving both GI and mental health symptoms is likely due to its ability to modulate immune responses, restore intestinal barrier function, and correct dysbiosis [51].

The positive effects of a LFD on psychological well-being appear to vary depending on the IBS subtype. In a post hoc analysis of the DOMINO trial, which compared the effects of a FODMAP-restricted diet to a spasmolytic agent (otilonium bromide) for improving IBS symptoms, only patients with IBS-D showed significant improvements in psychological well-being [55,56]. These included reductions in scores on the depression scale (PHQ-9), the generalized anxiety disorder questionnaire (GAD-7), and the somatization status (PHQ-15), highlighting the subtype-specific benefits of dietary interventions [56].

Interestingly, FODMAP-induced symptoms in IBS patients might be likely driven by altered brain responses to pain, highlighting the role of GBA dysfunction in symptom generation.

Jie Wu et al. study [57] investigated the mechanisms behind FODMAP-induced symptoms in IBS patients by examining their gut and brain responses to fructans administration compared to healthy controls (HC). In this randomized double-blind cross-over trial, participants received intragastric infusions of fructans (40 g/500 mL saline), glucose (40 g/500 mL saline), or saline (500 mL) while undergoing MRI scans of the brain and abdomen. Although both IBS patients and HCs showed similar increases in small bowel motility and colonic gas/volume following fructans intake, only IBS patients experienced significantly more cramps, pain, flatulence, and nausea after the fructans infusion. In these patients, brain regions associated with pain processing—such as the cerebellum, the anterior/mid cingulate cortex, the insula, and the thalamus—responded differently to fructans, and these responses were correlated with the severity of symptoms [57]. These results were later commented on by Halmos EP and Gibson PR [58], who emphasized that this study used high and unrealistic doses of fructans that may not accurately reflect a typical dietary intake. Thus, they recommended that the scientific community consider GBA dysfunction and use more rational doses of FODMAPs in research to better represent their role in IBS symptomatology [58]. Table 2 provides a summary of studies on the LFD and its impact on anxiety and depression.

## 5. Discussion

**Figure 2:**
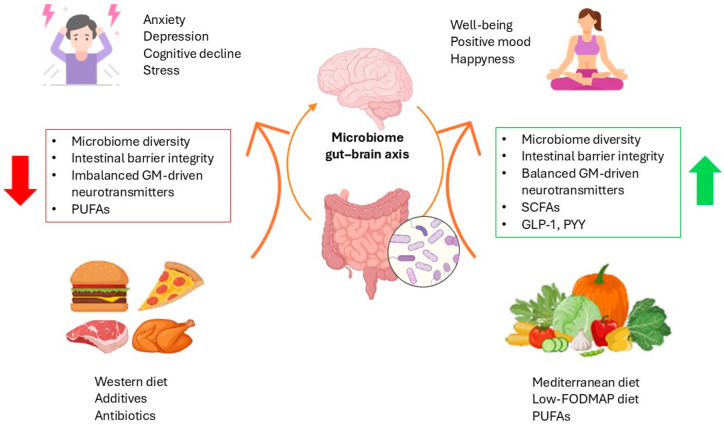
Impact of diet on the gut–brain axis: effects on gut health, mental well-being, and psychopathology. Abbreviations. PUFAs: Polyunsaturated fatty acids; GM: Gut microbiota; SCFAs: Short-chain fatty acids; GLP-1: Glucagon-like peptide-1; PYY: Peptide YY; FODMAP: Fermentable oligosaccharides, disaccharides, monosaccharides, and polyols. The arrows represent the bidirectional interaction within the gut-brain axis.

The gut–brain axis (GBA) is a complex communication network connecting the GI tract with the CNS. This bidirectional system encompasses neural, hormonal, immune, and microbial pathways, with the GM playing a crucial role in regulating these interactions. Diet significantly influences the GBA, affecting both gut health and mental well-being, as well as the levels of psychopathology (Figure 2).

The role of FODMAPs in modulating the GBA represents a promising approach for managing both the physical and psychological aspects of digestive health conditions. By implementing dietary interventions like the LFD, clinicians can assist patients in gaining better control over their symptoms, which may lead to improvements in overall mental health and quality of life.

Limitations and considerations regarding the LFD must be carefully examined. Although the short-term benefits of an LFD in improving GI symptoms and certain aspects of mental health are well documented, its long-term effects on the gut microbiome and overall health remain unclear. The diet’s restrictive nature may also lead to nutritional deficiencies or adverse changes in the GM if not properly managed. Therefore, ongoing research and careful clinical monitoring are necessary to ensure that patients achieve balanced and sustainable outcomes. Looking ahead, one promising area of research is the combination of the LFD with the MD [59]. Both dietary approaches have demonstrated significant benefits for gut health and mental well-being, but integrating them could offer a synergistic effect that enhances therapeutic outcomes. MD, known for its anti-inflammatory, antioxidant, and prebiotic properties, could complement and bolster the effects of the LFD, which primarily focuses on reducing GI symptoms by limiting fermentable carbohydrates. This integrative approach could not only enhance the management of IBS but also contribute to improved mental health outcomes, addressing both the physical and psychological dimensions of the GBA. Future research should explore the long-term effects of combining these dietary interventions, assessing their impact on gut microbiota, intestinal permeability, immune function, and mental health to fully understand the potential of this dietary synergy.

## 6. Conclusions

The interplay between diet, the GM, and mental health is increasingly recognized as a pivotal factor in managing various forms of psychopathology, such as anxiety and depression, particularly when they are linked to IBS. Specific dietary interventions, such as the Mediterranean diet and the LFD, have shown significant potential in improving mood and overall psychological well-being by positively influencing the GM composition. These dietary patterns not only alleviate GI symptoms but also enhance emotional resilience and overall happiness.

## Figures and Tables

**Table 1:** Summary of studies on dietary interventions and their impact on psychopathology.

Authors (Year)	Study Design	Sample Size	Population	Type of Intervention	Psychopathology Assessed	Principal Key Findings
Martin SE et al. (2022) [18]	Pilot study	20	Adult	High fat/protein vs. high carb/fat diet	Anxiety, depression	Higher fat/protein intake linked to reduced anxiety/depression, inversely related to GM diversity
Lin PY et al. [24]	Meta-analyses	3318	Depressive patients and control subjects	Omega-3, Omega-6 PUFAs	Depression	Lower levels of EPA, DHA, and total *n*-3 PUFA in patients with depression
Selvaraj R et al. (2022) [27]	Systematic review	N/A	Adult	General Dietary Habits	Depression	Systematic review finds healthy diet, avoiding junk foods, fast foods, and high meat intake may lower the risk of developing depressive symptoms
Jacka FN et al. (2017) [33]	RCT	67	Depressive patients and control subjects	12-week modified Mediterranean diet (Australian)	Moderate to severe depression	Significant reduction in depressive symptoms in diet group compared to control
Parletta et al. (2019) [36]	RCT	95	Adults with self-reported depression	MedDiet for 3 months and fish oil supplements for 6 months vs. social groups fortnightly for 3 months	Depression and mental health	Higher MedDiet adherence, particularly with fish and vegetables, reduced depression and improve mental health
Cabrera-Suárez BM et al. (2024) [37]	RCT	196	Recovered depressed patients	2-year MD with extra virgin olive oil supplement vs. usual care	Depression recurrence	Significant improvements in depressive symptoms after 4, 8 and 20 months in MD compared to usual care, despite no differences in depression recurrence risk
Staudacher HM et al. (2024) [38]	RCT	48	IBS and mild or moderate anxiety and/or depressive symptoms	6-week Mediterranean diet vs. habitual diet	Anxiety, depression	MD improved GI symptoms and depression compared to habitual diet
Luo Y et al. (2023) [39]	Meta-analysis	83.533	Adult	Fermented dairy foods	Depression risk	Fermented dairy consumption is associated with reduced depression risk

Abbreviations: N/A: Not applicable; IBS: Irritable bowel syndrome; RCT: Randomized controlled trial; DHA: Docosahexaenoic acid; EPA: Eicosapentaenoic acid; PUFA: Polyunsaturated fatty acids; MD: Mediterranean diet; MedDiet: Mediterranean-style diet.

**Table 2:** Summary of characteristics of low-FODMAP studies and its impact on anxiety and depression.

Authors (Year)	Study Design	Sample Size	Population	Diet Intervention	Outcome Measures	Principal Key Findings
Ledochowski et al., 2000 [43]	Observational	53	Fructose malabsorbers	Elimination of some FODMAPs	Mood and depressive symptoms, QoL, meteorism, stool-frequency	Elimination of fructose and sorbitol improved depressive symptoms
Piacentino D et al., 2016 [44]	RCT	75	IBS patients	4-week LFD, LFD gluten-free diet and a control diet	SCL-90-R, intensity of abdominal bloating/pain, frequency of bloating/pain	Both LFD improved in bloating, abdominal pain, anxiety, and phobic anxiety scores
Eswaran et al., 2017 [45]	RCT	92	IBS-D	4-week LFD vs. mNICE	QoL, anxiety, depression, work productivity and sleep quality	Significant improvements in health-related QoL, anxiety, and activity impairment
Staudacher et al., 2017 [46]	RCT	104	IBS patients	4-week sham diet vs. LFD, along with a placebo or multistrain probiotic	GI symptoms, stool frequency and consistency, QoL, faecal PCR and 16S rRNA sequencing	Adequate symptom relief with LFD. Co-administration of probiotic increased numbers of Bifidobacterium species
Harvie et al., 2019 [47]	RCT	50	IBS patients	12-week LFD	IBS-SSS, QoL, microbiome analysis	Reduction of IBS-SSS and increase of QoL. No change in microbiome when during LFD
Pedersen et al., 2014 [48]	RCT	123	IBS patients	6-week LFD vs. Lactobacillus rhamnosus GG and non-intervention control group	IBS-SSS and QoL	No significant difference in improvement between LFD and probiotics
Ustaoğlu T et al., 2024 [49]	Clinical Trial	52	Female IBS patients	6-week LFD vs. LFD with Lactobacillus rhamnosus	IBS-SSS, anxiety, and depression, QoL	Significant reductions in IBS-SSS, anxiety, and depression scores and increase of QoL in both groups
Kortlever et al., 2019 [50]	Prospective observational	111	IBS patients	6-week LFD	Long term QoL, GI symptoms, anxiety/depression, fatigue, sleep quality, and happiness	Improvement of long-term QoL, GI symptoms, reduced fatigue and anxiety/depression, and increased overall happiness
Prospero L. et al., 2021 [51]	Clinical Trial	20	IBS-D patients	12-weeks LFD	IBS-SSS, QoL, SCL-90-R, QPF/R, SF-36, sugar absorption test, indican and skatole evaluation, serum and faecal zonulin levels, FABP, DAO, IL6, Il10	Improvements in GI symptoms and psychological states, with enhanced intestinal permeability
Jie Wu et al., 2022 [57]	RCT	26	Female IBS patients vs. HC	Fructans infusion vs. glucose/saline	Bloating, fullness, nausea, cramps, pain, flatulence, bowel motility and ascending colonic gas	IBS patients experienced unique brain responses to fructans correlated with GI symptom
Halmos EP and Gibson PR 2022 [58]	Letter	N/A	N/A	Commentary on fructans study [57]	N/A	Emphasized the need for realistic doses of FODMAPs in research

Abbreviations: N/A: Not Applicable; RCT: Randomized controlled trial; IBS: Irritable bowel syndrome; IBS-D: IBS with diarrhea; HCs: Healthy controls; GI: Gastrointestinal; FODMAP: Fermentable oligosaccharides, disaccharides, monosaccharides, and polyols; LFD: Low-FODMAP diet; mNICE: modified diet recommended by the National Institute for Health and Care Excellence; QoL: Quality of life; IBS-SSS: IBS symptom severity scoring system; SCL-90-R: Symptom checklist-90-revised; QPF/R: Psychophysiological questionnaire; SF-36: 36-item short-form health survey; FABP: Fatty-acid binding protein; DAO: Diamine oxidase; IL: Interleukin.
